# Dr. Forbes's New Translation of the Second Edition of Laennec on Auscultation

**Published:** 1828-01-01

**Authors:** 


*Traité de l'Auscultation Mediate, &c. A Treatise on the Diseases of the Chest, and on Mediate Auscultation.* Translated from the French of R. T. H. Laennec, M.D. with copious Notes, and a Memoir on the Life of the Author, by John Forbes, M.D. senior Physician to the Chichester Infirmary, &c. Second Edition. One volume, 8vo. pp. 722, with Plates, and a Head of the Author. London, 1827.

Eight years have now elapsed, since we first called the attention of the profession, in this country, to the treatise of M. Laennec, which we characterised as one of the most valuable productions that had appeared in the present age. In doing this, we rested our opinion chiefly on its pathological merits, having then had but little personal experience of the application of the author's discovery to the diagnosis of thoracic diseases. Since that period, however, we have had abundant opportunities of verifying the truth, and of appreciating the value, of *mediate auscultation;* and we can now as confidently bear testimony to the value of the diagnostic part of M. Laennec's work, as we then did to its pathological merits.

As, on the occasion alluded to, we entered very fully into the pathological views of the author,* and again took up the subject in noticing Dr. Forbes's translation of the first edition, we do not now mean to give any thing like a formal *analytical* review of the work before us. And we the more readily come to this determination, seeing that, in its present improved form, it must soon be in the possession of every medical man, who is really desirous of making himself acquainted with a class of diseases, which he is called upon to treat more frequently, perhaps, than any other; and his successful treatment of which must of course, depend no less on his knowledge of their nature, than on his powers of distinguishing them from each other. Our principal object, in this notice, will be to point out to our readers the very material improvements and additions with which the present edition has been enriched by the distinguished author and able translator.

The work has now attained all the perfection which it ever can receive from the hand of M. Laennec, whose premature death we had lately the melancholy task of recording. He just lived to see the present edition published, the completion of which, with his other arduous professional avocations, seems clearly to have hastened his end. The death of M. Laennec has deprived the profession of one of its most talented and most zealous members, and the medical school of Paris of one of its brightest ornaments; and this, too, at a period of life, when, judging from his past labours, so much might still have been expected from him.†

The present edition possesses many and great advantages over the former. It is much enlarged and improved in every respect. The arrangement is infinitely superior, the pathological descriptions more complete, the diagnostic signs are more correctly stated, and the limits and practical utility of

* See vol. II. of this Journal, Jan. 1820, when we occupied upwards of thirty closely-printed pages, in giving a condensed analysis of the first edition.

N.B We were a good deal surprised at a singular mis-statement in Dr. Forbes's dedication, wherein he gives Dr. Clark the credit of being the *first* to promulgate the work of Laennec in this country. Our analysis of it came out several months before Dr. Clark's notes were printed.

† Dr. Forbes has prefixed to his translation an interesting sketch of the author's life, containing a history of his studies, an accurate list of his numerous writings, and an estimate of his private and professional character. He was only in his 45th year when he died. It is singular that none of Laennec's biographers have noticed his genius for, and even practical knowledge of, mechanics; though it was, in all probability, this turn of mind which led to the discovery of the stethoscope.

mediate auscultation more clearly defined. The *treatment* of the different diseases is also added in the present edition; and the work must now be considered as a very complete treatise on the pathology, diagnosis, and method of cure, of the diseases of the thoracic viscera:—

"The original treatise," says Dr. Forbes, and in this sentiment we entirely accord, "will remain an imperishable monument of the genius and industry of the author; and the discovery of which it treats, will entitle him to a distinguished rank among the benefactors of mankind. As a standard work on the pathology and diagnosis of diseases of the chest, it is not only without an equal, but may be considered as almost perfect in its kind. Much, no doubt, will hereafter be discovered that will modify and improve the delineations of disease which he has left us, but their great outlines must remain unalterable as nature itself."*

Besides the treatment and general improvements which we have noticed, numerous additions have been made to the original materials of this work, which we shall rapidly glance at. In the very important chapter on "*Inflammatory Affections of the Mucous Membrane of the Bronchia*," the sections on *pituitous*, *dry*, *latent*, *suffocative*, and *symptomatic catarrh*, and also on *hooping-cough*, are either altogether new or greatly enlarged. The chapter on *Dilatation of the Bronchia* is also much augmented, that on *Croup* is entirely new. The subsequent chapters on *Bronchial Hæmorrhage*, *Bronchial Polypus*, *Ulcers of the Bronchia*, &c. are materially enlarged. The dissertation on the structure of the lungs, introductory to the diseases of these organs, contains much new matter. The chapters on *Atrophy* and *Hypertrophy* of the lungs are new; that on *Emphysema of the Lungs* is much enlarged, and one of the most interesting in the treatise. The chapter on *Peripneumony* is materially improved, and really constitutes a complete and elaborate monography of pulmonic inflammation. The article *Phthisis* is greatly enlarged and also materially improved; and, like many others in the work, constitutes a beautiful specimen of accurate pathological research. Before the publication of the work of Luis, this monography of consumption was unrivalled; even now, with the aid of the translator's copious annotations, derived from the work of Luis and many others, it is probably still the best memoir on the subject; certainly it is, beyond all question, the best in the English language. The chapters on *Diseases of the Pulmonary Vessels*, and on *Nervous Affections of the Lungs*, are mostly new.

To the second grand division of the work, which treats of

---

* Translator's Preface, p. 1.

the heart and its appendages, the additions are less important, though this part is also much enlarged and improved. The new sections on *nervous affections of the heart and arteries* are interesting, and conclude the work.*

Thus much for the original: with respect to Dr. Forbes' translation, it has our most unqualified praise. He has not only given a complete version of the present edition, but has enriched the original matter with copious and valuable notes; partly from the stores of his own observation, and partly selected from the older authors, both foreign and English, whose works Laennec had overlooked. He has also been enabled to supply much valuable matter from the works of Andral, Luis, &c. which have appeared since Laennec's work was printed. Dr. Forbes's acquaintance with the German and Italian languages has also enabled him to draw upon the medical literature of these countries on many occasions. And it may be stated generally, that where Laennec's account of any disease is incomplete, Dr. Forbes has either added what was wanting, or has indicated the sources whence more full information may be obtained. On the treatment, which certainly is not equal to the other parts of the work, though still containing many practical remarks well deserving the attention of the English physician, the notes supply much valuable information. They are evidently the produce of a mind well stored with knowledge, and possessing a sound judgment to direct its practical application. Dr. Forbes, with talents of no ordinary stamp, evinces in all his writings the highest degree of candour and liberality: attached to no sect, and free from prejudice and bias of every kind, he willingly grants to each man the merit that is his due, to whatever age or nation he may belong. Though fully impressed, we observe, with the value of the great improvements introduced into medicine by modern pathologists, and more especially by the French, we are happy to see that he does not, on that account, overlook the stores of sound practical knowledge to be found in the writings of the older authors, particularly those of our own country, and which are too much neglected in the present day. The notes, in truth, contain a vast mass of important information, which renders the translation a much more valuable work than the original. The whole treatise deserves the careful perusal of every medical man, but we would particularly call the attention of the younger

---

* In comparing the translation with the original, we perceive that Dr. Forbes has made several transpositions of chapters and sections which appear judicious.

members of the profession to the chapters on bronchial diseases, which are treated in a masterly manner. The dry and latent forms of catarrh demand especial attention, as being little attended to—we had almost said, entirely overlooked in this country; though the neglect of them often leads to the most distressing and even fatal consequences. They very frequently lay the foundation of those interminable coughs, dyspnœas, asthmas, &c. which embitter the remaining period of thousands of lives, which they eventually shorten. They also complicate many other diseases, and render them much more difficult of cure, for instance, fever, pneumonia, pleurisy, &c. The complication of these, and indeed of all the forms of chronic catarrh, with an irritated condition of the mucous membrane of the gastric system, forms one of the most important subjects, in a therapeutical point of view, with which we are acquainted. The following excellent practical remark shews that the morbid connexion in question has not escaped Dr. Forbes's observation. After noticing the use of copaiva and similar medicines recommended by authors for the cure of catarrhal affections, he observes—

> "A circumstance not much noticed by these writers, and which renders all such plans of treatment nugatory, is the frequent co-existence, especially in old persons, of a similar condition of the mucous membrane of the upper portion of the alimentary canal. In this complication, every thing stimulating, whether as food or medicine, is decidedly injurious; while the most marked benefit is derived from such mild regimen as the obvious condition of the membrane indicates."*

The article on pleurisy is altogether excellent, and the treatment good. In one point of practice, in which we find Dr. Forbes differs from the author, (who prefers cupping to the application of leeches,) we decidedly agree with M. Laennec. In cupping the operation is much less tedious and harrassing to the patient, the quantity of blood abstracted can be accurately regulated, and the exposure of the chest, which we hold to be of great consequence to guard against, is much more easily avoided.

In the treatment of peripneumony our views do not accord so fully with those of M. Laennec. He carried the use of tartar emetic to a great extent, and we must say, in our opinion, without much judgment or discrimination. The following is the author's own account of his practice.

> "As soon as I recognize the existence of pneumonia, if the patient is in a state to bear venesection, I direct from eight to sixteen ounces of blood to

---

* P. 76—note.

be taken from the arm. I very rarely repeat the bleeding, except in the case of patients affected with disease of the heart, or threatened with apoplexy, or some other internal congestion. More than once I have even effected very rapid cures of intense peripneumonies without bleeding at all; but, in common, I do not think it right to deprive myself of a means so powerful as venesection, except in cachectic or debilitated subjects. In this respect M. Rasori does the same. I regard blood-letting as a means of allaying, for a time, the violence of the inflammatory action, and giving time for the emetic tartar to act. Immediately after bleeding I give one grain of the tartar emetic, dissolved in two ounces and a half of cold weak infusion of orange-leaf, sweetened with half an ounce of syrup of marsh-mallows or of orange-flowers; and this I repeat every second hour for six times; after which I leave the patient quiet for seven or eight hours, if the symptoms are not urgent, or if he experiences any inclination to sleep. But if the pneumonia has already made progress, or if the oppression is great, or the head affected, or if both lungs, or one whole lung, is attacked, I continue the medicine uninterruptedly, in the same dose and after the same intervals, until there is an amendment, not only in the symptoms, but indicated also by the stethoscopic signs. Sometimes even, particularly when most of the above unfavourable symptoms are combined, I increase the dose of the tartar emetic to a grain and a half, two grains, or even two grains and a half, without increasing the quantity of the vehicle. Many patients bear the medicine without being either vomited or purged. Others, and indeed the greater number, vomit twice or thrice, and have five or six stools the first day; on the following day they have only slight evacuations, and often, indeed, have none at all."*

We have no doubt of the powers of antimony in subduing pulmonary inflammation; but we are equally satisfied that it requires to be exhibited with circumspection, and with due regard to the peculiar character of each individual case. The general use of it, however, in large doses, and in the indiscriminate manner in which M. Laennec and the followers of Rasori employ it, we regard as both injudicious and dangerous; we, therefore, perfectly coincide in the judicious comments which Dr. Forbes has made on this subject, and to which we beg to refer the reader.† Pneumonia is often complicated with irritation and congestion of the abdominal viscera, and requires a corresponding modification of treatment. In this combination will in truth be found a key to the very contradictory statements regarding the utility of emetics, purgatives, &c. in

* The report which M. Laennec gives of the success of this treatment appears great, though certainly some of the most observant pupils who attended his clinic were not equally satisfied on this point with the professor himself; and we confess the result of our enquiries on the subject incline us to the opinion of those, who think that M. Laennec deceived himself in estimating the extent of the success obtained by his tartre stibié, and underrated, perhaps, the effects of the venesection which was premised in those cases in which the antimony appeared to be most successful.—*P.* 250, *Translation.*

† See notes on the treatment of peripneumony, by the translator.

pneumonia. Every physician of observation must have remarked the comparative facility of curing simple pulmonary inflammation, and the great difficulty of managing it when complicated with the pathological state to which we allude. When all is sound below the midriff, things turn out well, often under the most unpromising circumstances; while the reverse too frequently obtains when the abdominal viscera are in an unhealthy state. The translator has some long, and rather learned notes on the employment of antimony—we make one selection for our present purpose.

"In respect of the administration of the emetic tartar in pneumonia complicated with gastric disorder, I should say that it requires the utmost caution generally, and the greatest attention to each particular case, in order to guard against producing great mischief by it. In many of those cases of gastric complication recorded by Stoll, Reviere, Hellis, and others, where the affection consists rather in a loaded condition of the stomach, duodenum, and liver, and a vitiated state of their respective secretions, than in inflammation or high irritation of the mucous membranes, no doubt the emetic tartar may be valuable, at all events, as an emetic: but when evident signs of the other condition of parts exist, we cannot administer this remedy without imminent danger of augmenting the evils we are attempting to alleviate. That even in these latter cases, the emetic tartar is sometimes useful, I do not deny; but I believe Broussais' opinion on this point will be found to be generally correct: he says, speaking of emetic tartar in simple inflammatory affections of the stomach, 'leur effet est incertain dans les cas légers; et dans les graves, ils sont toujours dangereux, parcequ' ils ne manquent jamais d'augmenter l'inflammation qu' ils n'ont pas réussi à enlever,' (*Prop. de Med.*) But my principal object at present is, to call the attention of practitioners to the frequent co-existence of gastric affections with pneumonia in this country, and to point out the absolute necessity, in such cases, of treating *both* diseases at the same time. In the simple disease we shall generally find our bleedings from the arm, and our tartar emetic, according to the French phrase, *heroic;* while, in the complicated affection, we shall find these means, if not injurious, at least inefficacious, if we fail to attack the gastric affection with leeches to the epigastrium, saline refrigerants, mucilaginous diluents, &c. and if we do not forbid the ingestion of purgatives and other irritants, at least for a season."

In the truth of these remarks we perfectly accord. Dr. Forbes, in another note, gives the results of his own experience of tartar emetic, which are, upon the whole, favourable, and will be so, we doubt not, in the hands of those who use the same judgment and discrimination in the employment of it that Dr. Forbes does.

On *asthma*, in its various forms, we find much most valuable matter in this edition; and, taken in conjunction with the preceding accounts of *dry catarrh* and *emphysema of the lungs*, affords more precise information respecting the pathology of asthma than is to be met with in the writings of all preceding authors. Indeed, after a careful study of this part of the work, we are disposed to believe, with the translator, "that we have such additional light thrown upon this disease, *(asthma,)* that it may henceforth, in a great measure, be considered as raised from the obscurity of hypothesis into the light of rational pathology." In the following remarks by Dr. Forbes, on the treatment of asthma, we perfectly concur.

"Among the remedies that best deserve notice in asthma, I would mention a mild and spare diet, residence in a more temperate climate, and warm bathing. The first of these measures will be found very beneficial in cases complicated with gastric irritation; the two last are especially indicated in that class of cases which date from the disappearance of cutaneous eruptions under the use of powerful external applications. This method of cure is, I am convinced, the fruitful source of many internal irritations and inflammations, and, among others, of bronchitis and asthma. Although the doctrine of repulsion may be deemed by some theorists somewhat absolute, I feel assured that its truth will be assented to by most observant practitioners of experience. I therefore consider it my duty to caution the student against a practice, which accords too well with the energetic empiricism so much in favour in this country, not to be readily adopted from analogy, even if not inculcated by positive precept."—*(Note, p.* 418.*)*

But to notice all the valuable articles in the volume before us would be to go over the table of contents. In no other work is the same accurate and comprehensive information, on the various subjects of which our author treats, to be found. It is truly a classical production, not only to be read but studied;—a work of reference, in short, which almost every practitioner might advantageously recur to while the disease is under his observation. By comparing the descriptions of Laennec with nature, the clinical student more especially, to whom the work is invaluable, will learn to estimate the graphic delineations of the author, while he will greatly increase his knowledge of disease, and improve his powers of observation at the same time.

The second division of the work, which treats of diseases of the circulating system, is less perfect than the other; but Dr. Forbes's notes and references go far to supply its deficiencies. We make a single extract from one of the former, because it contains a practical admonition of great importance.

"I would therefore lay it down as a valuable practical rule in chronic affections of the heart, that previously to having recourse to any remedies intended to act directly on it, we ought to be assured that the digestive organs are in a healthy state—that their mucous surfaces are free from irritation—their vascular system not morbidly distended, and that the liver is performing its secretory function freely and regularly."*

---

* Note, p. 687. We recommend the careful perusal of the whole of this note to every medical man. In the conluding part of it, as on many other occasions, the translator bears testimony to the benefits derived by the profession from the labours of Broussais.

We hold this to be a golden rule, as well in chronic affections of the heart, as in other chronic diseases.

But we must leave this part of the work, to make a few remarks on the method of diagnosis recommended in it. We shall not occupy our reader's time, by enquiring into the causes which have retarded the progress of *mediate auscultation.* They are much the same which have operated against the introduction of every important discovery connected with the advancement of medical science, from the time of Harvey downwards. We are ready to grant, also, that there are some circumstances which operate against the application of the stethoscope, peculiar to itself; but even these have been greatly magnified, and will be entirely disregarded, as the conviction of its practical utility becomes established.

The application of auscultation to the diagnosis of diseases is surely a philosophical experiment. No good reason can be certainly adduced, why we should not avail ourselves of the information to be obtained through the medium of the sense of hearing, in increasing our powers of detecting the nature of diseases. And if we can shew that there is a most important class of diseases, the nature and seat of which it is often impossible to discover by other means, while it is yet of great practical importance to distinguish them, not only from one another, but in their different stages, we think it will be difficult for the opponents of auscultation to adduce satisfactory reasons for rejecting it.

It is notorious, and matter of daily occurrence, that great and important errors are committed in the diagnosis of thoracic diseases; most, if not all, of which might be obviated by means of auscultation.

"I will go so far as to assert," says Laennec, "and without fear of contradiction from those who have been long accustomed to morbid dissections, —that, before the discovery of Avenbrugger, one half of the acute cases of peripneumony and pleurisy, and almost all the chronic pleurisies, were mistaken by practitioners, and that, in such instances as the superior tact of a physician enabled him to suspect the true nature of the disease, his conviction was rarely sufficiently strong to prompt and justify the application of very powerful remedies."*

Now the greater number of medical practitioners, in this country, are precisely in the same state as the French practitioners were before the time of the justly celebrated Corvisart, who first called the attention of his countrymen to the discovery of Avenbrugger; for percussion is scarcely more frequently employed in England than the stethoscope; and yet it is

* Introduct. p. 2.—*Trans.*

justly characterised by Laennec as "one of the most valuable discoveries ever made in medicine."

"By means of it (he adds) several diseases which had hitherto been cognizable by general and equivocal signs only, are brought within the immediate sphere of our perceptions, and their diagnosis rendered both easy and certain."*

Even in our hospitals, percussion, we believe, is little used, though we will venture to affirm that, before a physician has made trial of it in half a dozen cases, he shall be so satisfied of its practical utility, that he will experience surprise, and, it may be, regret, that he had not had recourse to it sooner. But the information to be derived from *mediate* auscultation is far more extensive and accurate, though, as Laennec justly observes, the combination of the two methods becomes still more useful than either singly.

Too much has been required of the stethoscope by those who were prejudiced against it: if it did not tell every thing, it was considered as doing nothing; and the sins, moreover, of the auscultator have been, not unfrequently, visited upon the instrument. It has been said, too, most incorrectly, that mediate auscultation has been brought forward as a substitute for all other diagnostic signs. M. Laennec never had such an intention; and Dr. Forbes takes particular pains, in his preface, and other parts, to define the value, and limit the reliance to be given to auscultation. In a note on the article phthisis, he observes,

"It is only by combining the practice of auscultation with the faithful observation of symptoms, and by studying the results obtained from both sources, with a reference to the pathology of the disease, that we can hope to attain such a certainty of diagnosis as can satisfy a philosophical mind."

This is the true light in which auscultation should be employed—not as a substitute for, but as an addition to, the means we already possess of discriminating diseases. So far from leading us to relax in our researches, it tends rather to sharpen the intellect, by giving us a new interest in the disease. Having, by means of auscultation, ascertained the precise nature and stage of the disease, we may, by close observation of the morbid phenomena which accompany it, greatly improve our general diagnostic power. Before we know the exact pathological condition of an organ, the general symptoms produce but a vague and feeble impression on our mind, because we know not to what particular lesion they are referable. But this once ascertained, every thing connected with the case acquires a vast increase of value and interest, and every obser-

* Introduct. p. 3.—*Trans.*

vation we afterwards make forms a step towards a positive knowledge of the symptoms which characterise the disease. We have, thus, in mediate auscultation a new source of power, which, being well applied, may ultimately enable us to dispense with the very means by which we acquired it. It has also been used as an argument against the stethoscope, that the knowledge communicated by it comes too late for useful practical purposes. This has arisen chiefly from mediate auscultation having been too much associated with phthisis, and from its value in other diseases having been estimated by its practical utility in consumption; while, in general, and used alone, it is comparatively of little use in this disease: but the reverse holds true in other diseases, and the great practical utility of auscultation rests on the fact of diseases being discovered by it in their more early stages, when the most observant physician is doubtful of their nature. In the latent periods of disease, auscultation will often convert our bare suspicions into certainty, and enable us to apply our means of cure at a period when they avail the most. In pneumonia, in bronchial diseases, and in some affections of the heart, the disease not unfrequently remains latent during the greater part of its progress, or is only accompanied by symptoms which do not enable the practitioner to detect its nature till it is too late—sometimes not until it is disclosed by the dissecting knife! This is still more strikingly the case in some fevers, accompanied with oppression of the nervous system, in which extensive disease of the lungs occurs, without being accompanied with the usual indications. This is the case in a remarkable degree, also, in that fatal termination of many surgical diseases, by supervening, but masked, peripneumony, as noticed by some of our best surgical writers, Mr. C. Bell, Mr. Guthrie, &c. as referred to by Dr. Forbes in a note, p. 238. In the case of young children, also, where we are deprived of half the information on which we are accustomed to form our opinions, auscultation becomes the only diagnostic measure on which we can safely depend. On examining bodies, carried off apparently by fever, at all ages, extensive inflammation of the lungs is often discovered, though, during the life of the patient, the existence of such an affection was scarcely suspected, and the detection of which, in due time, might have been the means of saving him. Yet this post-mortem examination is of little utility to practitioners in general, because it does not shew them how such cases are to be detected on another occasion:—On him, however, who can call auscultation to his aid, the discovery of such a case produces a very different effect. He is not likely to lose another patient under similar circumstances; or, at least, he will not do so, without being aware of the nature of the fatal disease, if he is not able to prevent its becoming so, which will often be the case. The stethoscope thus communicates the same certain kind of information which, in too many cases, we obtain only at the dissection-table: with this difference, that, in the former case, we have the disease still under our observation, and may turn our knowledge to the advantage of our patient; while, in the latter case, we can only regret that we did not know what the dissecting-knife has disclosed to us, while our patient was yet alive.

The difficulty of acquiring a knowledge of the application of the stethoscope has been urged as a reason against its general employment; but against what part of medical knowledge might not the same argument be used? It is much easier to become acquainted with mediate auscultation, for all useful practical purposes, than it is to become acquainted with the indications afforded by the pulse. The sense of hearing no doubt differs in acuteness in different individuals, just as the other senses do, and there can be no question, that some will, by one week's practice of the stethoscope, acquire more tact in its application, than others will in several weeks, or even months; but we are well assured, that there is scarcely any one whose acoustic organs are so dull, that he may not, in a very short period, acquire sufficient proficiency in its use, to enable him to distinguish the principal diseases of the thoracic viscera from each other, so as to render his practice much more satisfactory to himself, and infinitely more beneficial to his patients. We can speak strongly on this subject from practical experience. Even in a negative point of view, we have felt much indebted to the stethoscope, for setting our mind at ease in some of those nervous affections, which simulate organic disease of the respiratory organs, and, at times, produce such alarming symptoms, as to embarrass the most experienced practitioner. But the greatest enemy to mediate auscultation, after all, we fear, is indolence, which, as Dr. Forbes well remarks, (note, p. 78) "is a potent and prevailing advocate, even with the most active. We readily persuade ourselves, that what is very troublesome to do may be left undone, with little detriment to ourselves or others; and that an easy substitute is an adequate substitute." For our own parts, we are thoroughly convinced, that, in the present state of our knowledge, there is no adequate substitute for auscultation in discriminating the various important diseases to which the thoracic viscera are obnoxious, and which, it is to be feared, too often lead to a fatal result, because their nature is misunderstood, or because they are not recognised till the period has gone by at which our art is of much avail.

Deeply impressed with the great advantages which the profession—which mankind—may derive from the general introduction of auscultation, (under which term we comprehend percussion, and immediate, as well as mediate, auscultation*) we think it our duty to urge our professional brethren, and above all those who are at the head of our public medical institutions, to give the measure a fair trial.† Those who hold the highly responsible office of instructing the rising members of the profession, should reflect on the mischief they may be doing by neglecting auscultation, even if it might claim only one half the praise which we are satisfied it deserves. All we ask for is a fair trial, and we fear not the decision of every candid and honest mind. Too often, we fear, has the measure been condemned after a trial altogether inadequate, and we readily sympathise in the indignant feelings expressed by Dr. Forbes, on the contemplation of such unphilosophical proceedings.

"When, therefore, we hear," he says, "as we sometimes do, that certain persons have *tried the stethoscope*, and abandoned it upon finding it useless or deceptive; and when we learn, on enquiry, that the *trial* has extended only to the hurried examination of a few cases within the period of a few days or weeks; we can only regret that such students should have

---

* Much of the information afforded by the stethoscope may be obtained more directly by the immediate application of the ear to the chest of the patient; but, independently of various objections to this method, it does not generally afford the same accurate information as the stethoscope, and, in several situations, is not at all applicable. We, therefore, strongly recommend the beginner to commence his observations with the stethoscope; the substitution of the ear is afterwards easy, and often convenient—not so the reverse. Every person desirous of making himself acquainted with the nature, the value, and method of employing auscultation, should read with care M. Laennec's *Preliminary Essay on the physical Diagnostics of the Diseases of the Chest, &c.* On *Percussion*, which our author has treated too briefly, the reader is referred to the translation of Avenbrugger by Dr. Forbes, or Corvisart; the former has some interesting cases attached to it, illustrative of the utility of mediate auscultation.

† In Dublin, where, as it seems to us, medicine is cultivated more as a science, and with greater zeal, especially in their public institutions, mediate auscultation, as might be expected, is in far greater estimation than on this side St. George's Channel. We had occasion to make pretty copious extracts, in our last number, from a valuable paper in the fourth volume of the Dublin Hospital Reports, by Drs. Graves and Stokes, the principal object of which was to "prove the utility of the stethoscope in the *diagnosis* and *treatment* of thoracic diseases." The remarks of these gentlemen on hydrothorax we recommend especially to the attention of the opponents of auscultation.

been so misdirected, or should have so misunderstood the fundamental principles of the method. No conclusion, deduced from such attempts—I cannot dignify them with the term experience—can have any weight with those qualified to judge of the matter; they can only be added to the heap of *false facts*, as they have been called, with which medicine, and indeed every department of human knowledge, is overlaid, and which are the characteristic and ready offspring of minds too feeble to be habitually conversant with general principles, and too narrow to embrace all the more important relations of the objects of their enquiry."*

We believe the stethoscope has been condemned on slighter trials than Dr. Forbes alludes to, and often without any trial at all. But we have already extended this article beyond the limits which we had assigned to it, though we shall neither consider our own, nor our reader's time, as wasted, if our remarks on auscultation shall tend to its more general adoption.

Before concluding, we think it our duty to say one word on the literary merits of Dr. Forbes's translation. We have read it with care, and have no hesitation in pronouncing it to be one of the best, if not the very best, translated medical works in our language. The sense of the author is given in correct and perspicuous language, while the style is at, the same time, much more condensed than the original.† It is evidently the work of a scholar, thoroughly acquainted with the language he is translating, as well as with that in which he is writing. We would recommend Dr. Forbes's work as a model for translators. We now bid our authors farewell; grateful for the information we have derived from their labours. To M. Laennec it is a last farewell; but his translator, we trust, we shall soon meet again on the literary arena, satisfied that we can never do so without increasing our knowledge.‡

---

* Translator's Preface, p. viii.

† By this condensation, and using a large page, Dr. Forbes has comprehended in one moderate sized volume, what was spread over two in the original, a great advantage in a book of reference.

‡ As it is our intention, in the Review department of our Journal, for the future, to single out, as far as is possible, *particular subjects*, in the works reviewed, and reflect on them all the collateral lights of experience and research, with the view of forming, in fact, a series of monographs on the principal diseases to which the human frame is liable, so we will again revert to the work now superficially reviewed, and take occasionally one of the more important topics therein contained for our text. By this plan we hope not only to render our Review department still more valuable than it has hitherto been, but we shall thereby defy all attempts at anticipation of our labours by the "flying artillery" of the present times. In each HALF-MONTHLY fasciculus or part of our next and succeeding numbers, we shall give one very extensive, or two moderately sized *eclectic articles* of the above description, which we hope to render a very important feature in our Journal, and a very great improvement in the Review department.

